# Single-cell RNA sequencing and kidney organoid differentiation

**DOI:** 10.1007/s10157-023-02359-5

**Published:** 2023-05-20

**Authors:** Kohei Uchimura

**Affiliations:** Division of Nephrology, University of Yamanashi, 1110 Shimokato, Chuo, 409-3898 Japan

**Keywords:** Kidney organoid, scRNA-seq, Kidney disease model, Collecting duct biology, Maturation

## Abstract

Since 2015, Japanese researchers have made great progress in developing a method to differentiate human pluripotent stem cells (hPSCs) into kidney organoids. Protocols have been established to produce increasingly complex three-dimensional (3D) structures, which are used as a human kidney disease model and adapted for high-throughput screening. During this period, single-cell RNA sequencing (scRNA-seq) technology was developed to perform a comprehensive analysis at the single-cell level. We have performed a comprehensive analysis using scRNA-seq to define how kidney organoids can be applied to understand kidney development and pathology. The structure of kidney organoids is complex and contains many cell types of varying maturity. Since only a few proteins and mRNAs can be identified by immunostaining and other techniques, we performed scRNA-seq, which is an unbiased technology that can comprehensively categorize all cell types present in organoids. The aim of this study is to review the problems of kidney organoids based on scRNA-seq and the efforts to address the problems and predict future applications with this powerful technique.

## Introduction

Rodents have been used as kidney disease models in a variety of preclinical trials for decades, but it is often difficult to apply the drugs that effectively treat kidney diseases in rodents to humans. One major reason is that rodents and humans have a different kidney development and genomic regulation [1]. Further complications can be attributed to the fact that different strains of mice differ in their susceptibility to kidney disease [2–5]. Japanese researchers have been developing kidney organoid differentiation methods from hPSCs, which has generated great enthusiasm among researchers, to better understand the pathology of human-specific kidney diseases [6–8]. Although the kidney organoid field has developed rapidly, the diversity and maturation state of organoid constituent cells have yet to be investigated further. Conventional strategies, such as histology, immunofluorescence, and quantitative PCR, characterize a limited number of markers on kidney organoids. These experimental methods have low throughput and can measure only a few markers selected by the researchers. Bulk RNA sequencing allows for a more comprehensive analysis of organoid gene expression [9]; however, it is impossible to assign the gene expression to a specific cell type because it reflects the average expression profile of all cells in the organoid as a lump. Hence, further research is needed to determine what cell types exist within organoids, their degree of differentiation, and how to improve the differentiation protocols, which can be confirmed via scRNA-seq. Because scRNA-seq allows unbiased and comprehensive gene expression profiling of thousands of single cells without any prior knowledge, we used this technique to assess the cell types and gene regulatory networks that comprise the kidney organoid [10]. This review highlights how scRNA-seq is being used to apply kidney organoids to regenerative medicine and human kidney disease modeling.

## Kidney organoids

**Fig. 1:**
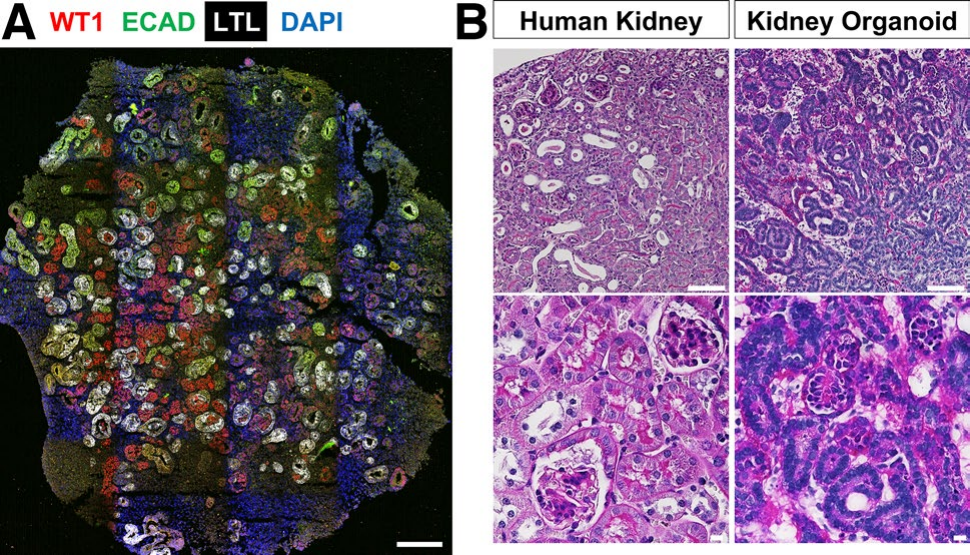
Immunofluorescence and histology of kidney organoids. **A** Immunofluorescence staining of markers for podocytes (WT1 in red), proximal tubule (LTL in white), and distal tubule (ECAD in green) in a whole kidney organoid. Scale bar, 500 μm.** B** Periodic acid-Schiff–stained sections of the human kidney (left) and kidney organoid (right). Scale bar, 500 μm (upper), 10 μm (lower)

The generating kidney organoids protocols have been established from hPSCs based on the knowledge of mammalian kidney development [11, 12]. 3D culture technology has enhanced the ability of embryonic and adult stem cells to self-organize and form multi-cellular tissues, enabling differentiation into kidney tissue. Thus, hPSCs are induced to differentiate into primitive streak, intermediate mesoderm, and subsequently nephron progenitors that ultimately self-organize by manipulating the Wingless-related integration site (Wnt) fibroblast growth factor (FGF) and transforming growth factor b (TGF-b) pathways [13]. Mature kidney organoids contain about one hundred nephrons composed of glomeruli, properly segmented tubules, and interstitial cells (Fig. 1) [14]. The ability to culture kidney organoids from patient-derived tissue has allowed researchers to study human kidney development and pathology. Regenerative medicine research using hPSCs has two goals: one long-term goal is to create transplantable kidneys, and the other realistic goal is to establish disease models and apply them to drug discovery research. Disease modeling has been particularly well developed, for instance, kidney organoids have been used to successfully model and drug screening for autosomal dominant polycystic kidney disease (ADPKD) [15], acute kidney injury (AKI) [16], and vascularization of the glomerular tuft [17].

## scRNA-seq

scRNA-seq was developed as a powerful technology to comprehensively explore transcriptional heterogeneity in large and diverse cell populations by measuring gene transcription in individual cells. A typical scRNA-seq experiment can involve profiling 10,000 cells. Each cell is analyzed by measuring the expression of approximately 10,000 different mRNAs from about 4000 different genes. Even in a simple experiment, 10,000 different mRNAs are detected from 10,000 cells, resulting in an output of 100 million data points. Several methodologies have been used for scRNA-seq, but they all share a common procedure: (1) single cell or single nucleus isolation, (2) cell lysis and RNA capture, (3) reverse transcription and amplification, (4) library generation, and (5) next-generation sequencing [18]. To date, scRNA-seq analysis has been performed on healthy kidneys, developing kidneys, kidney disease, and kidney organoids. These studies provide a better understanding of the mechanisms leading to kidney injury and the important pathways involved in kidney development.

## Comprehensive analysis of kidney organoids with scRNA-seq (Fig. 2): off-target cells [10]

**Fig. 2:**
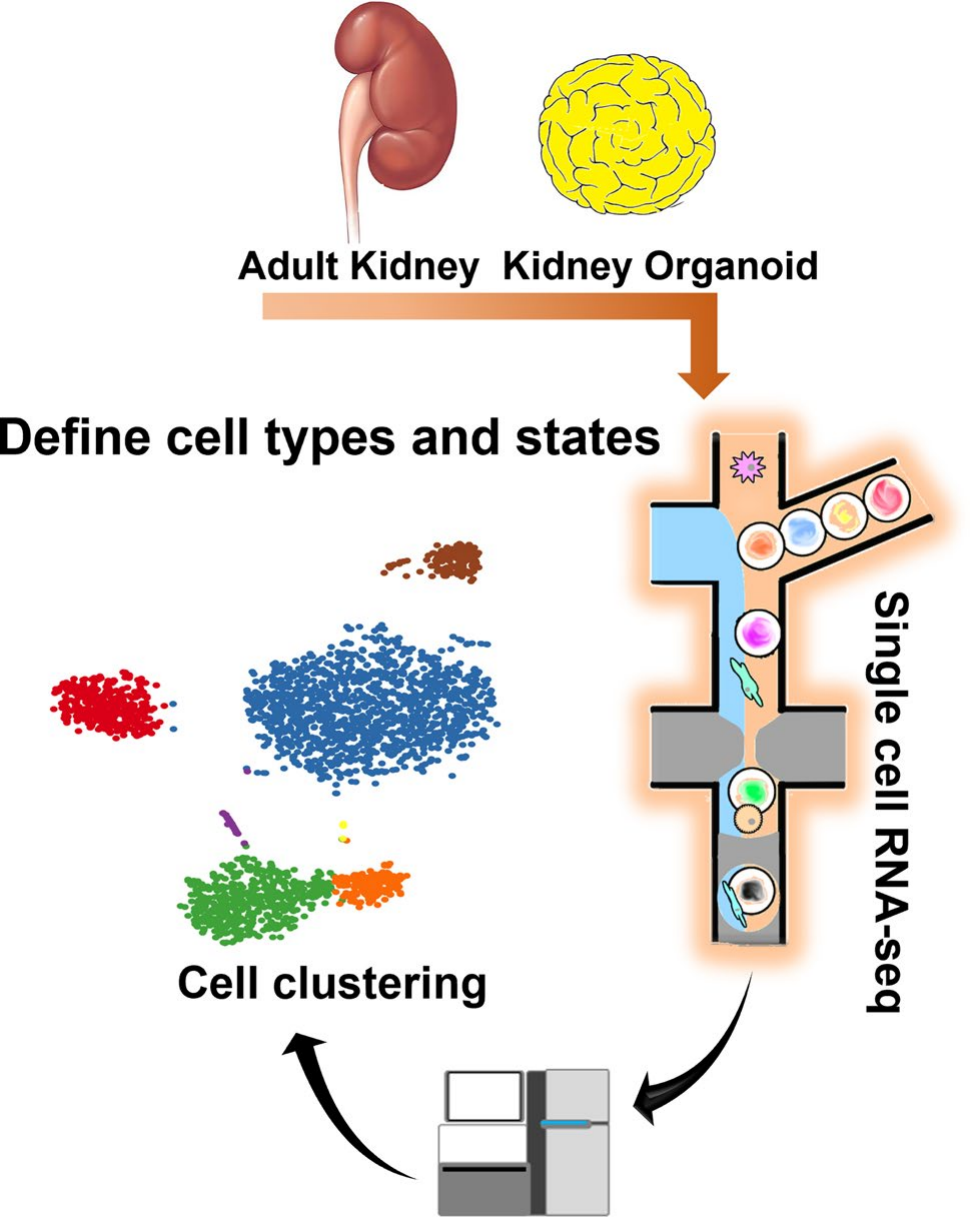
The scheme of scRNA-seq experiment and analysis

**Fig. 3:**
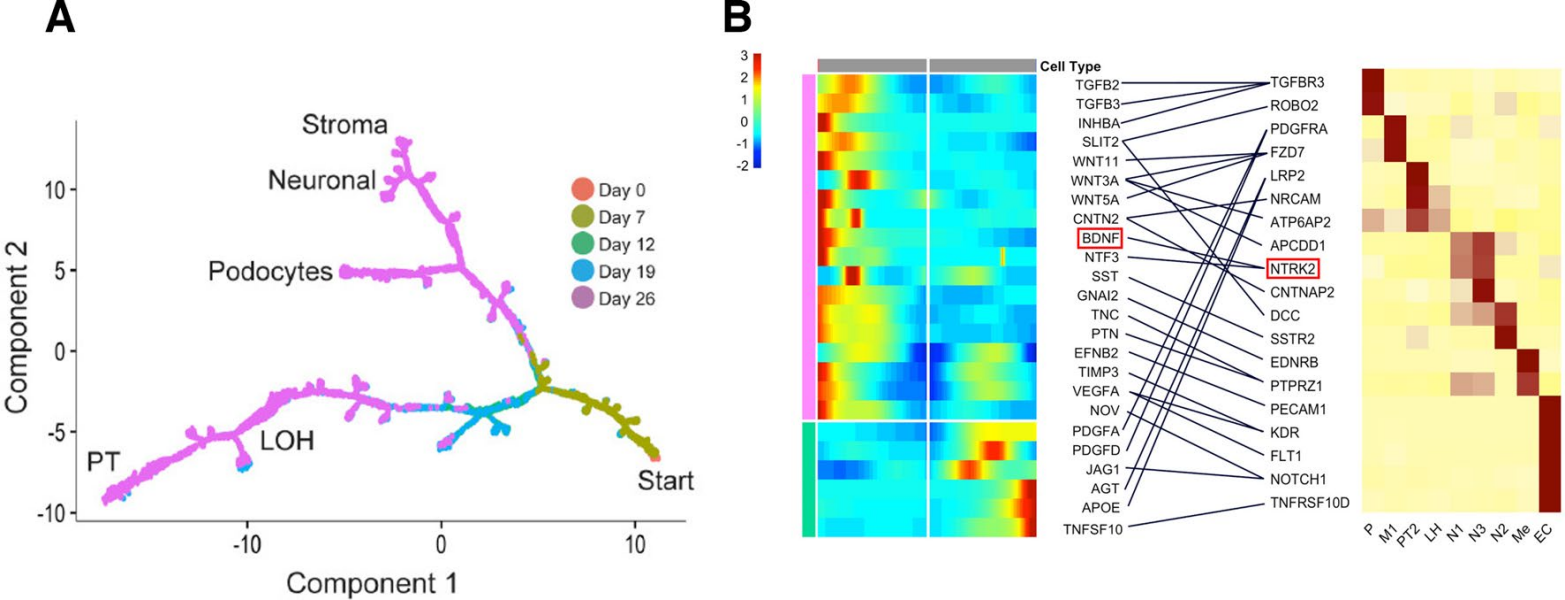
Pseudo-temporal ordering and ligand-receptor analysis [10]. **A** Ordering of scRNA-seq expression data based on the pseudo-temporal position along the lineage indicated a continuum of gene expression changes from iPSCs to differentiated cell types. **B** Heat map showing the kinetics of branch-dependent ligand expression identified by BEAM (Monocle2) and corresponding cell-specific receptor expression in day 26 organoids from the Takasato protocol. The analysis identified BDNF and its receptor NTRK2 as exclusively expressed in neurons

**Fig. 4:**
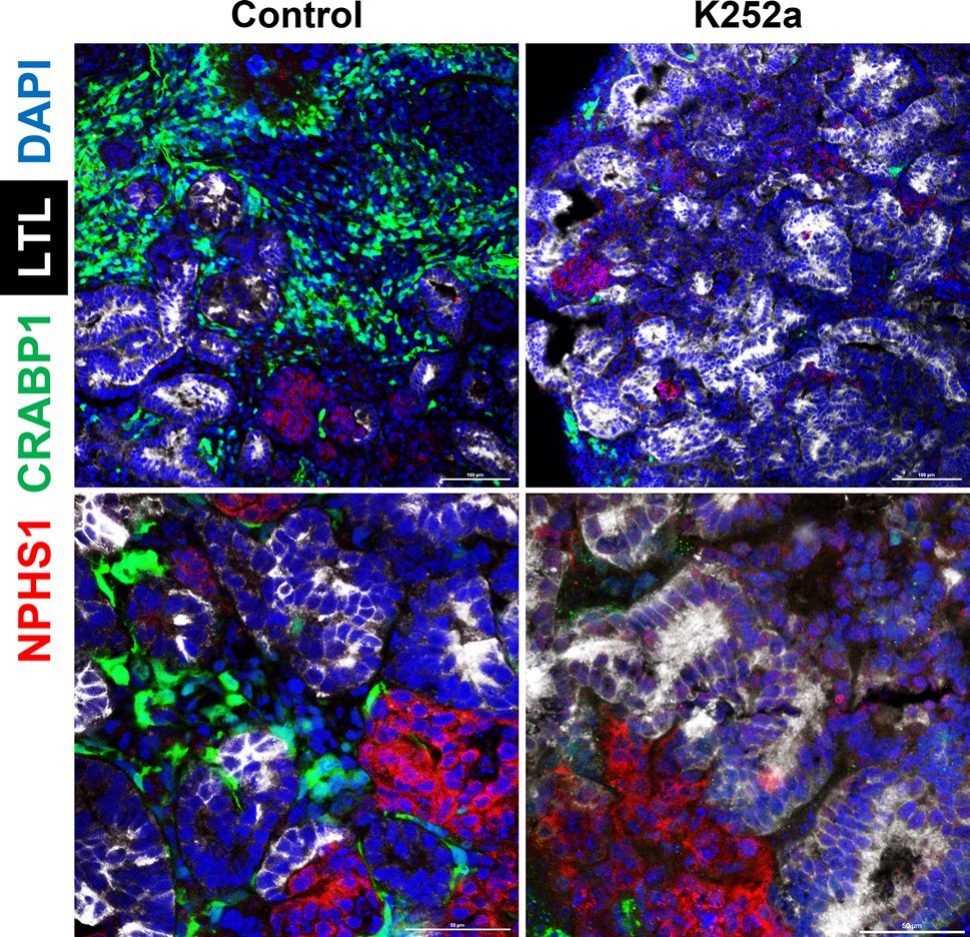
BDNF inhibition significantly reduces off-target neurons in the kidney organoid [10]. Immunofluorescence staining of markers for podocytes (NPHS1 in red), neurons (CRABP1 in green), and proximal tubules (LTL in white) in the kidney organoid. NTRK2 inhibitor (K252a) significantly reduced green off-target neurons (right)

We and several research groups have reported that kidney organoids contain cells not presenting in the real kidney, such as neurons, muscle cells, and melanocytes, regardless of the protocol employed [10, 19, 20]. These off-target cells interfere with normal kidney differentiation, resulting in differences from human kidney modeling. We have attempted to eliminate off-target cells based on the scRNA-seq data set. The pseudo-temporal sequence allows us to investigate changes in the signaling pathway and critical regulatory factors expressed when determining the cell fate decision. Adjusting the expression of these critical signaling pathways and regulatory factors at a particular time point can improve the differentiation protocol. We performed scRNA-seq over time during the induction of kidney organoid differentiation to eliminate uninvited off-target cells from the kidney organoids. Next, we examined gene expression changes at the point of differentiation in each cell lineage based on pseudo-temporal ordering. Ligand-receptor analysis revealed that the nerve growth factor brain-derived neurotrophic factor (BDNF) and its receptor, tropomyosin receptor kinase B (NTRK2), are strongly expressed only in neurons that have departed from the nephron progenitor cell lineage (Fig. 3) [10]. Since BDNF is known to be essential for neuronal survival and growth, we hypothesized BDNF-NTRK2 signaling might contribute to the differentiation of these off-target neurons [21]. Adding an NTRK2 inhibitor (K252a) to the differentiation protocol resulted in a 90% reduction in off-target neurons (Fig. 4) [10]. The application of ligand-receptor analysis can potentially reduce other off-target cells in various organ organoids, contributing to improved protocols.

## Lack of a mature collecting duct

Until a recent genetic lineage analysis indicated that ureteric bud (UB) derivatives are present in some kidney organoids, other investigators have been unable to identify a definitive UB lineage including principal cells (PCs) markers, such as aquaporin-2 (AQP2) as well as acid- and base-secreting intercalated cells (ICs) [8, 19, 22]. A rudiment of the kidney, the embryonic metanephros, develops by the mutual interaction of the metanephric mesenchyme (MM) and the UB [23, 24]. However, most protocols pursued MM lineage, including nephron progenitor cells, and have not achieved the induction of UB-derived mature collecting ducts. The interconnection between MM and the UB is an important step for kidney organoid formation. Taguchi et al. used separate protocols for the induction of MM and UB precursors, followed by recombination, which significantly improved the collecting duct architecture [25].

## Immature cell states

Although the human kidney develops over about 200 days [26], kidney organoid protocols generally last only about 25 days. Thus, the organoid component cells are expected to be immature compared with adult kidney cell types. Little et al. reported that kidney organoids are as immature as first-trimester human fetal kidneys as confirmed by bulk RNA sequencing [7]. We performed a similar comparative analysis using the scRNA-seq approach [10]. The results confirmed that, as previously reported, kidney organoids are immature with gene expression patterns comparable to those of fetal kidneys. The scRNA-seq data revealed transcriptional differences between organoid-derived cells and adult kidneys, with deficient expression of maturation nephron markers in kidney organoids. In contrast, developmental marker expression was much higher in kidney organoid. Researchers worldwide are working to generate more mature kidney organoids [27, 28].

## New protocol

### Collecting duct maturation

**Fig. 5:**
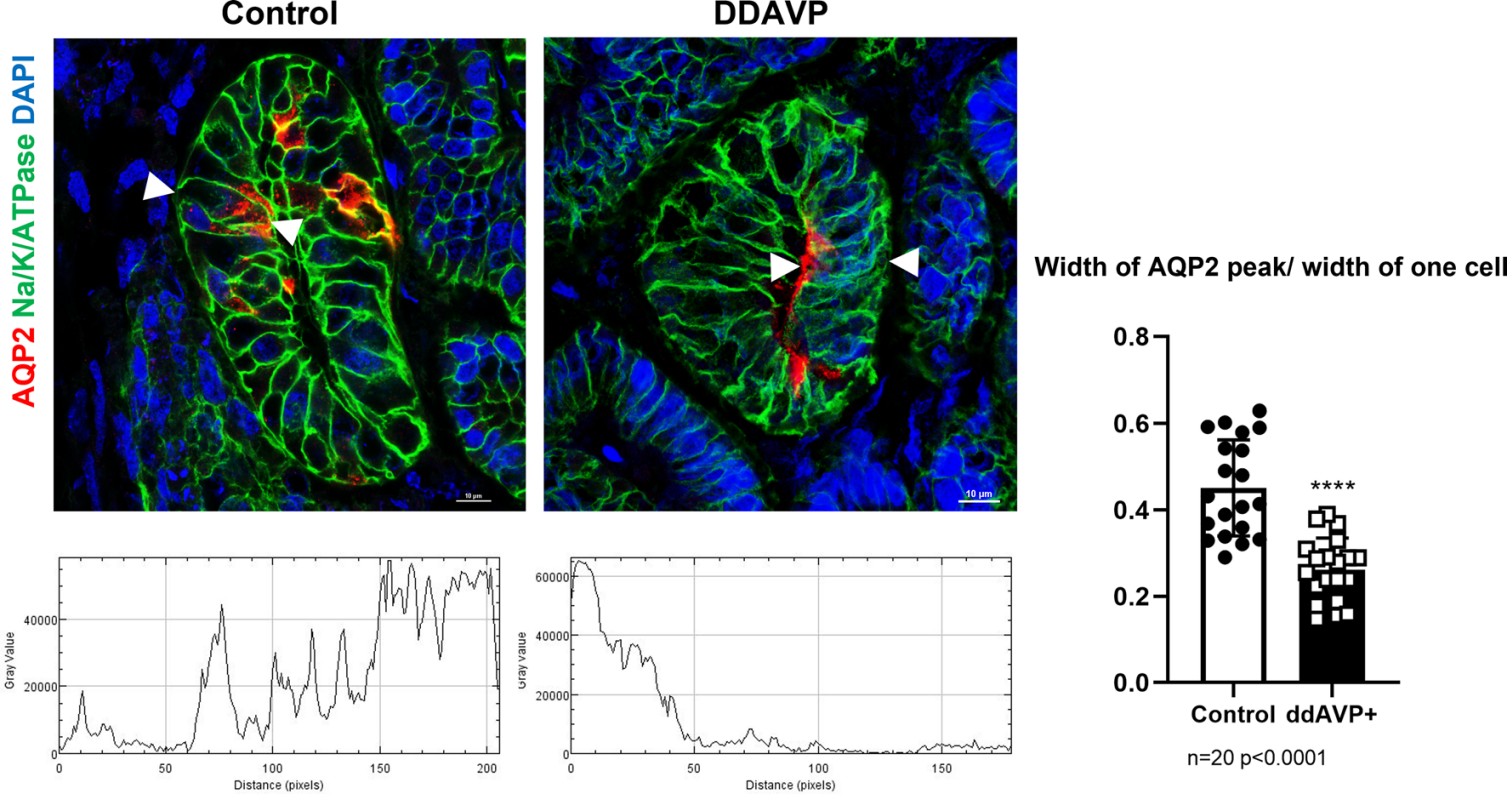
Vasopressin-stimulated AQP2 insertion in the kidney organoid [31]. Fluorescence images from organoids incubated with vehicle or 10 nM AVP for 3 h after 24 h washout. The AQP2 protein translocate to the apical surface (arrowhead) after AVP stimulation. Bar graph quantifies translocation

Mae et al. reported a method for UB induction [29], followed by Tsujimoto et al. in the same research group, who reported a separate induction method for mesoderm progenitor cells based on their work from hPSCs [30]. After combination, the organoids contain glomeruli and tubules as well as collecting ducts and could become vascularized when implanted in mice. We also established a different protocol for the individual induction of both MM and UB-like progenitors from hPSCs [31]. The combination of these progenitor cells results in assembly collecting duct-like structures, with the addition of the endocrine hormone vasopressin and aldosterone, develop both PCs and ICs that express terminal differentiation markers. We were able to observe the physiological activity of the vasopressin-induced membrane translocation of AQP2 proteins (Fig. 5)[31]. We could also detect urothelial cells for the first time and could shift the ratio of PCs and ICs by modulating the Notch signaling.

### Tubular injury [31]

**Fig. 6:**
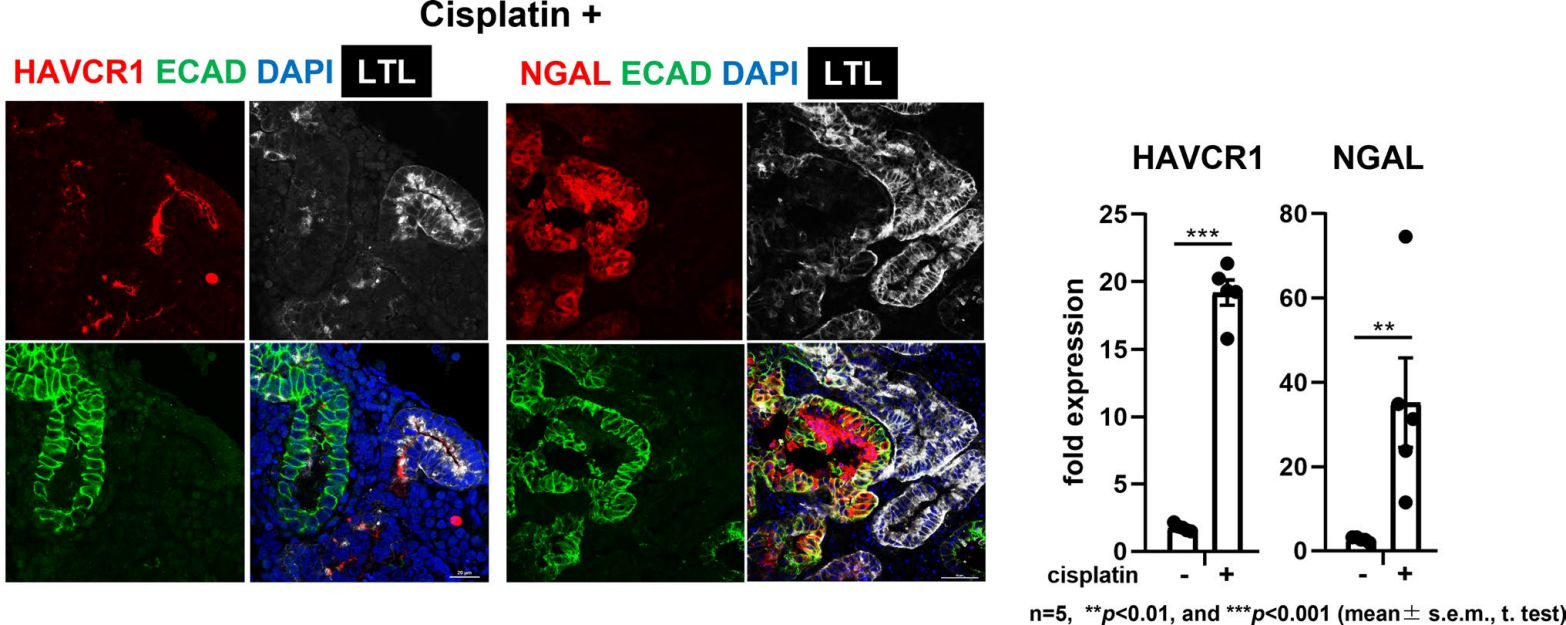
Injury responses in the kidney organoid [31]. Fluorescence images and qPCR (*n* = 5) showing the upregulation of the proximal tubule injury marker HAVCR1 in the LTL-positive proximal tubule after 48 h cisplatin exposure and distal tubule injury marker NGAL in the E-cadherin-positive distal tubules

Several research groups have reported the modest induction of apoptosis and kidney injury markers, such as hepatitis A virus cellular receptor 1 (HAVCR1), in response to tubular toxins [7, 8, 32] specifically at the proximal tubule [33]. Neutrophil gelatinase-associated lipocalin (NGAL) is also kidney injury biomarker, expressed in both the thick ascending limbs and collecting ducts, but to our knowledge has not been detected in kidney organoids [34]. We have confirmed that the epithelial toxicant cisplatin induced both NGAL and HAVCR1 in our new organoids. As expected, we could verify that the HAVCR1 protein is located apically in LTL-positive proximal tubules, and NGAL protein is specifically induced in ECAD-positive distal tubules (Fig. 6)[31].

## Disease models using kidney organoids

The use of kidney organoids for disease modeling presents several challenges. Freedman et al. found that CRISPR/Cas9 knockout of podocalyxin causes defects in the junctional organization [15] and failure to assemble microvilli and lateral spaces [35] between developing podocytes, resulting in failure of junctional migration. They also showed that deletion of the PKD1 or PKD2 genes leads to giant cysts formation from the kidney tubules, reproducing human polycystic kidney disease [15]. Mutations in IFT140 have been implicated in human nephronophthisis (NPHP)-associated ciliopathies. Kidney organoids generated from patient-derived iPS cells with a compound heterozygous mutation in IFT140 recapitulate the phenotype of NPHP-associated ciliopathy, including shortened club-shaped primary cilia [36]. Nishinakamura et al. established the iPS cell from patients with NPHS1 missense mutations. They demonstrated that genetic correction could rescue the defect in slit diaphragm formation in NPHS1 mutant podocytes [37]. Morizane et al. applied kidney organoids to a pathological model for AKI to CKD as well as genetic disorders [38]. They found that repeated cisplatin treatment–induced tubular atrophy and fibrosis were associated with a reduction of the Fanconi anemia complementation group D2 (FANCD2) expression in gH2AX-positive tubular cells. A similar inverse correlation between FANCD2 abundance and tubular injury severity was also observed in the human kidney biopsy samples, indicating a variable severity of interstitial fibrosis and tubular atrophy.

## Drug screening using kidney organoids

Little et al. demonstrated that organoid-derived glomeruli maintain marker expression after prolonged incubation and are suitable for toxicity screening. They demonstrated toxicity of doxorubicin using kidney organoids, inducing glomerular fragmentation in a dose-dependent manner similar to that observed in congenital nephrotic syndrome [36]. McMahon et al. developed a platform for differentiating thousands of miniature kidney organoids consisting of one or two nephron-like structures in each organoid [38, 39]. They use this platform to identify a potent new inhibitor of cyst growth in the organoid models of ADPKD. The screening of a library of 247 protein kinase inhibitors identified several protein kinase inhibitors that potently inhibited cyst growth without suppressing overall organoid growth or development. One of these hits, the quinazoline derivate QNZ, exhibited a low IC50 of 5 nM for both PKD1 and PKD2 cell lines. They use kidney organoids to identify QNZ as a potent new inhibitor of cyst growth in ADPKD models.

## Current limitations and prospects

Organoids have significant advantages compared with classical cell cultures, but also have definite disadvantages compared with the original organs, besides immaturity, off-target cells, and lack of a collecting duct. Organoids are limited in the amount they can proliferate without causing cell death due to the lack of blood flow. Transplantation is the only way to induce blood vessels into the glomerulus. In addition, hiPSC-derived organoids implanted under the mouse kidney capsule showed signs of growth, integration with the host circulatory system, and further maturation, including glomerular basement membrane formation, maturation of the slit diaphragm in podocytes, and enrichment of brush border in proximal tubular cells [40–46]. Furthermore, since organoids lack immune cells, they are unsuitable for studying processes that require the critical elements of human physiology, such as the inflammatory responses invariably accompanying many nephropathies [47].scRNA-seq is a powerful technique used to analyze the gene expression at the single-cell level. This technique allows researchers to investigate cellular heterogeneity, identify novel cell types, and track the differentiation of cell populations over time, providing insights into developmental processes and disease progression. However, the scRNA-seq still has some limitations, such as the possibility that cells that are difficult to single-cell isolate may not be recovered (dissociation bias) and the changes in the gene expression owing to stress during the processing, which require caution in interpreting the data. Additionally, the scRNA-seq shows only the changes in the gene expressions. It is necessary to confirm the changes in the final product, protein expression by other methods. Recently, new technologies such as “single cell multiomics analysis,” which adds information from epigenetics analysis to transcriptome analysis and “spatial transcriptomics,” which integrates location information on tissue sections, have been applied to kidney research.

## Conclusion

The kidneys are complex organs that are composed of a wide variety of cells that maintain fluid and electrolyte homeostasis and remove metabolic waste products. Conventional cell culture methods remain insufficient to fully recapitulate renal physiology and pathogenesis underlying complex kidney diseases. Differentiation protocols have been developed to simulate kidney formation in vivo, but kidney organoids still present some limitations. The application of patient iPSC-derived kidney organoids provides a better understanding of the mechanisms in kidney maturation, injury, and repair, but there is still a need to modify current organoid protocols by employing new technologies such as 3D bioprinting and organ-on-a-chip to facilitate glomerular vascularization and integration of the collecting duct system into the organoid. Powerful technological advances like kidney organoids and scRNA-seq are expected to further advance kidney disease research via therapeutic target identification, toxicity prediction, and disease modeling.
